# Effects of Different Metals on Properties and Friction and Wear of Composite Materials

**DOI:** 10.3390/polym14214545

**Published:** 2022-10-26

**Authors:** Wei Li, Yihui Chen

**Affiliations:** 1 Shandong Hanggong Taifeng Braking Systems Holding Co., Ltd., Weifang 262700, China; 2 Weichai Power Co., Ltd., Weifang 262550, China; 3 College of Electromechanical Engineering, Qingdao University of Science and Technology, Qingdao 266061, China

**Keywords:** end face of internal mixer, corrosive wear, wear and tear, performance of mixed rubber

## Abstract

With the vigorous development of the automobile industry, the rubber industry has also made continuous progress. As necessary mixing equipment in the rubber industry, the internal mixer is required to undertake a lot of constant work for a long time, which inevitably causes wear to the internal mixer. On the one hand, the wear of the metal on the end face of the internal mixer will lead to an increase in the gap between the inner mixing chamber and the end face, which will lead to material leakage, affect the material ratio of the rubber mixture, and ultimately affect the performance of the rubber mixture. On the other hand, the wear of the end metal of the internal mixer is an increasing process, and the tiny metal particles of the end metal will be incorporated into the rubber mix along with the mixing process, affecting the performance of the rubber mix. At the same time, the disassembly and repair of the internal mixer are complex, and the end face maintenance is difficult. Therefore, finding a kind of end face metal with good wear resistance, long service life, and no influence on rubber compound performance is essential. This paper takes the end face metal of the internal mixer with severe wear as the research object. The wear degree of the metal after friction between MCYD-4 alloy, YW-15 alloy, wear-resistant stainless steel, tungsten carbide alloy, and the rubber compound is compared. The changes in the properties of the compounds after rubbing were investigated. The study found that the tensile tear properties, wet skid resistance, and rolling resistance of NR/BR composites differed when different end-face metals were selected for mixing, but the gap was small. When the end-face metal is YW-15 alloy, the NR/BR composites have the best dispersibility, the most robust tensile tear performance, the best wet-skid resistance, and minor rolling resistance. When the end face metal is the other three alloys, the physical and mechanical properties of the NR/BR composites are reduced to different extents. In this paper, starting from the actual working conditions, considering both abrasive wear and corrosive wear, the friction and wear between the rubber compound and the four kinds of metals commonly used on the end face of the internal mixer are studied. The metal that has little effect on the performance of the rubber compound and is the most wear-resistant was found. This paper is of great significance for improving production efficiency and prolonging the life of the internal mixer.

## 1. Introduction

With the successful development of the automobile industry, the rubber industry has also made continuous progress. As a piece of essential mixing equipment in the rubber industry, the internal mixer usually runs for a long time, which inevitably causes wear and tear to the mixer. On the one hand, the wear of the end face of the internal mixer leads to an increase in the gap between the mixing chamber and the end face, and the phenomenon of material leakage occurs, which changes the material ratio of the mixing rubber and affects the performance of the mixing rubber. On the other hand, the wear of the end face of the internal mixer is a continuous process of accumulation. The small metal particles worn off on the end face are integrated into the mixing rubber along with the mixing process, which also affects the performance of the mixing rubber. At the same time, the disassembly and repair of the internal mixer are complicated, and the maintenance of the end face is complex. Therefore, it is essential to explore a metal material with good wear resistance and long service time without affecting the performance of the mixed rubber for preparing the end face.

Qu [1] studied the friction and wear of high-chromium cast iron as a lining material and found that when silica was used as an abrasive, the surface coating of tungsten carbide could effectively improve its friction and wear performance, and reduce abrasive and cutting wear. Will Tp [2] established the formula for calculating the maximum pressure difference between the interior and exterior of the hydrostatic sealing ring by studying the relationship between the seal contact area and the maximum pressure difference between the interior and exterior of the end face. It was found that when the actual pressure difference was more significant than the calculated pressure difference by the formula, the seal end face may fail, resulting in substantial leakage. Metcalfe [3] studied the influence of temperature rise on sealing performance in the process of friction and wear of end face seal and deduced a simple calculation method for film thickness at different radii. Generally, the performance of materials was greatly affected by temperature, and materials were prone to large deformation under high temperature and pressure, thus changing their bearing capacity. Yelong Xiao [4] presented the design, characterization, and implementation of powder metallurgical metal-based friction materials for friction brakes. Yelong Xiao conducted an experimental study of the tribological behavior of friction materials under different conditions, with a particular focus on vacuum tribology. In addition, the corrosion resistance of the friction materials was evaluated by UV irradiation and atomic oxygen exposure tests. Hugh Spikes [5] investigated the effect of surface blasting on squeak generation, using a reciprocating configuration of balls sliding on a flat surface in two different arrangements with varying mechanical characteristics. The effect of using sandblasted surfaces with different roughness on the generation and evolution of squeaks was systematically investigated. Yahong Xue [6] established a three-dimensional finite-element model based on the classical Archard adhesive wear theory to study the failure process of self-lubricating plain bearings under oscillating wear conditions. The effect of surface blasting on noise generation was investigated by A.Y. Wang [7], who used a reciprocating spherical, flat sliding structure to systematically study the impact of blasting surfaces with different roughness on the age and evolution of cracks. Moustafa Mahmoud Yousry Zaghloul [8,9,10,11,12,13,14] has studied various nanocomposites and characterized their mechanical and wear properties.

As necessary mixing equipment in the rubber industry, the internal mixer is required to undertake a lot of continuous work for a long time, which inevitably causes wear to the internal mixer. The wear of the metal on the end face of the internal mixer will lead to the increase of the gap between the inner mixing chamber and the end face, which will lead to material leakage, affect the material ratio of the rubber mixture, and ultimately affect the performance of the rubber mixture. MCYD-4 and YW-15 alloy are relatively new alloys, and there is no application on the end face of the internal mixer. At the same time, the mixing process is accompanied by chemical and physical changes, and the mixing function of rubber and metal is highly complex. This paper used MCYD-4 alloy, YW-15 alloy, wear-resistant stainless steel, and chrome-plated alloy as research objects. The mixing process was accurately simulated, and the friction and wear phenomena between four end metals and rubber during the mixing process were studied. In this paper, many experiments have been carried out to measure the corrosion wear amount and abrasive wear amount of different metals, and the wear ratio has been calculated creatively. This paper is of great significance for prolonging the life of the internal mixer and improving rubber quality.

## 2. Experimental

### 2.1. Main Instruments and Equipment

The main instruments used for the experiment are shown in Table 1.

### 2.2. Experimental Formula

The materials used in this experiment are shown in Table 2.

The following were used: Butadiene Rubber 9000 (BR9000), China Hainan Natural Rubber Industry Group Co., Ltd. (Hainan, China); Natural Rubber (NR), China Hainan Natural Rubber Industry Group Co., Ltd. Company, (Hainan, China); Bis-[γ-(triethoxysilyl)propyl]tetrasulfide (TESPT), Antioxidant 4020, Zinc Oxide ZnO, Stearic Acid SAD, 1,3-Diphenylguanidine (DPG), accelerator CZ, and sulfur S, products of China Henan Longji Chemical Co., Ltd. (Hena, Chian); Silica115MP has a specific surface area of 115 m^2^/g, 7–40 nm particle size, and solid powder, Solvay Silica Co., Ltd. (Gunsan, Korea).

### 2.3. Mixing Process

In this experiment, the mechanical blending method was adopted. The temperature control device of the mixer was set, and the initial temperature was set to 40 °C. The mixing process is shown in Table 3.

When the time was 5 min, the rubber was discharged by the Hake mixer, and the obtained rubber was plasticized in the BL-6157 double high mill. First, the thin pass was carried out on the BL-6157 double high mill, and after five narrow passes, the rubber mixture was roll-wrapped. After roll wrapping, triangle roll wrapping and winding were carried out alternately on the double high mill, five times each. Finally, the roll distance of the double high mill was adjusted to 8 mm, the mixing rubber was cut, and the mixing rubber sample with a smooth surface was obtained.

### 2.4. Performance Test

#### 2.4.1. Rubber Processing Performance

The test equipment for the processing properties of rubber is RPA 2000.

**Figure 1:**
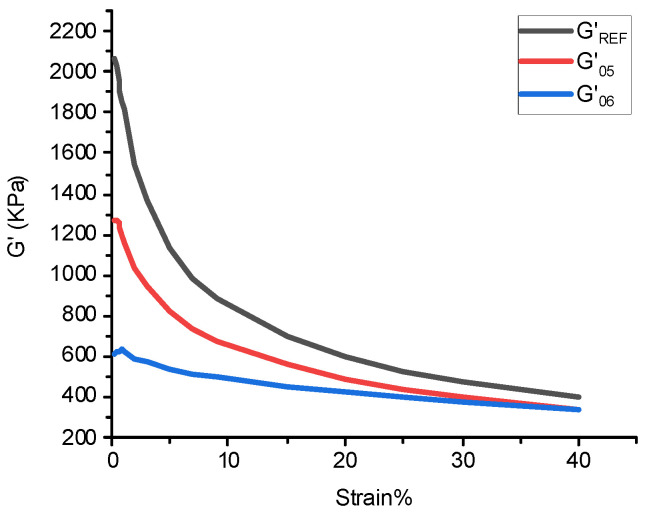
Distribution of silanization reaction degree.

(1) Payne effect: a sharp increase in the strain of the composite material and a subsequent sharp decrease in the dynamic modulus. The Payne effect is closely related to the cross-linked network inside the compound and reflects the particle dispersion of the composite [15,16,17,18].

(2) The silanization reaction index is calculated in Table 4 and Equation (1). The calculated image of the silylation reaction is shown in Figure 1.

(1) $$X=\frac{\mathrm{Area}\;\mathrm{of}\;\mathrm{silylation}\;\mathrm{zoon}}{\mathrm{Area}\;\mathrm{of}\;\mathrm{the}\;\mathrm{largest}\;\mathrm{silylation}\;\mathrm{region}}=\frac{\int G\prime REF(05)-\int G\prime S(05)}{\int G\prime REF(05)-\int G\prime S(06)}$$

#### 2.4.2. Tensile Tear Test

The purpose of this test is to study the properties of the compound after rubbing with different metals. To ensure the accuracy of the experiment, the mixed rubber tested in CSM was tested after rolling in the BL-6157 double-high mill [19,20,21,22,23,24,25].

Tensile and tearing experiments were conducted in the tensile testing machine, five tests were performed for each group, and the average value was taken.

#### 2.4.3. Vulcanizing Characteristics Test

A M-2000-AN non-rotor vulcanizer tested the vulcanizing characteristics. During the test, about 6 g of the rubber compound was put into the mold cavity of the non-rotor vulcanizer. The test temperature was 150 °C and the test time was 60 min. Finally, the vulcanization curve and parameters such as ML, MH, TS1, TS2, and T90 were obtained. The vulcanizing characteristics test of rubber can obtain the vulcanization time of rubber material and reflect the mixing effect of rubber material, which is an important test that must be carried out in rubber processing.

#### 2.4.4. Dynamic Mechanical Property Test of Rubber

The conditions of the rubber dynamic mechanical properties test were set as a temperature range of −65~65 °C, heating rate of 2 °C/min, maximum dynamic load of 40 N, and test frequency of 10 Hz, and the dual cantilever test mode was used under the protection of liquid nitrogen.

#### 2.4.5. CSM Friction and Wear Test

**Figure 2:**
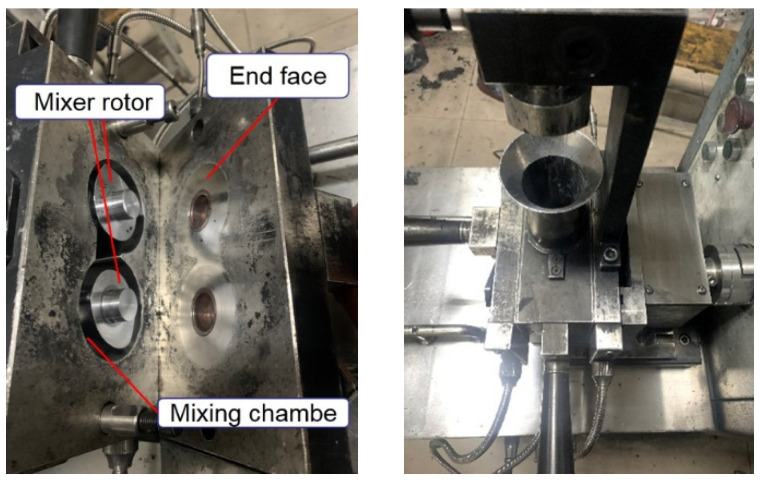
Hake internal mixer.

Figure 2 shows a Hake mixer whose rotor bulge is in contact with the concave surface of the sealing plate, and the sealing plate is pressed with the mixing chamber through-bolt fitting. There is a gap between the rotor and the mixing chamber. In the mixing process, the mixing rubber in the mixing section is in direct contact with the end face, which causes friction and wear to the end face metal. However, the metal volume of the end face of the mixer is too large to measure the wear quantity directly, so it is necessary to simulate the working process of the mixer to complete the metal wear test of the end face. The CSM friction and wear testing machine can affect the mixing process’s pressure, temperature, and speed. Therefore, the CSM friction- and wear-testing machine was used in this experiment to simulate the friction and model behavior between the mixing rubber and the end face metal and to reflect the wear amount of the end face metal by observing the wear amount of the metal grinding head.

After plasticizing in a BL-6157 double high mill, the mixed rubber with a smooth surface was cut to obtain cylindrical rubber samples with diameters of 100 mm and thicknesses of 8 mm. The CSM friction and wear were calibrated, and the parameters of CSM were set to the mixing process parameters. The pressure was set to 5 N, and the speed was set to 70 r/min. Metal wear was challenging to observe in a short time, so the CSM friction time was set to 60 min.

#### 2.4.6. Observation and Measurement of Three-Dimensional Morphology of Metals

The surfaces of the test samples were scanned by the 3D scanner (LEXT OLS5000, Olympus), and the change of morphology and volume of the samples before and after wear could be obtained.

#### 2.4.7. Dispersion Test

The vulcanized rubber was cut out of a new section with a cutter, and the team was tested with the dispersibility tester. The dispersibility value was obtained directly according to ASTM D7723.

## 3. Mechanism of Experiments

### 3.1. Mechanism of Silanization Reaction

**Figure 3:**
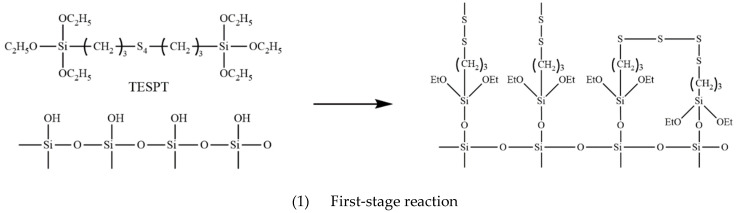

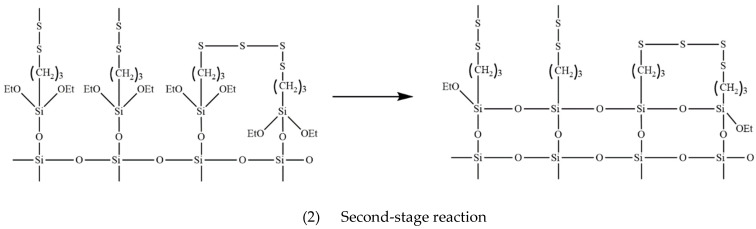
The silanization reaction.

The chemical name of TESPT is bis-[γ-(triethoxysilane) propyl] tetrasulfide. The silanization reaction of TESPT and silica is a critical chemical reaction in the mixing process, divided into two steps, as shown in Figure 3.

(1) First-stage reaction: the alkoxy group in TESPT is dealcoholized with the Si-OH group in Silica. The Si-OR group in TESPT is hydrolyzed to free the Si-OH group and dehydration condensation with the -oh group on the silica surface.

(2) Second-stage reaction: condensation reaction between adjacent silane coupling agents chemically bound to the surface of silica.

As shown in Figure 3, the silanization reaction produces ethanol and water. In the mixing process, the chamber is in a closed environment, and the products of silanization, ethanol, and water cannot overflow the internal mixer. During the mixing process, the shear and extrusion of the rotor lead to the continuous heating of the mixing chamber, which makes ethanol and water become high-temperature ethanol–water mixed vapor, which leads to friction and wears between NR/BR composites, and the metal on the end face becomes complicated. On the one hand, NR/BR composites wear the metal on the end face, and on the other hand, the corrosion wear is caused by high-temperature ethanol–water mixed vapor. Due to technical limitations, the quality of ethanol and water produced during the mixer mixing process cannot be measured. To ensure the accuracy of the experimental results, the mixing process was simulated on the CSM. The high-temperature mixing vapor was sprayed on the composite surface on CSM.

### 3.2. Friction Mechanism between NR/BR Composites and Metal

The friction between NR/BR composites and metal is mainly divided into abrasive and corrosion wear. This study is mainly carried out from these two aspects.

(1) SiO_2_ particles have the characteristics of easy agglomeration, high hardness, and irregular surface, and the accumulation of SiO_2_ particles is the leading cause of abrasive metal wear. Due to different metals’ surface hardness and wear resistance, abrasive wear caused by the accumulation of SiO_2_ particles is another characteristic. The experiments in this study are analyzed from this perspective.

(2) Corrosive wear on metal by high-temperature ethanol and water vapor mixture.

## 4. Results and Discussions

### 4.1. Dispersion Analysis

#### 4.1.1. Payne Effect

**Figure 4:**
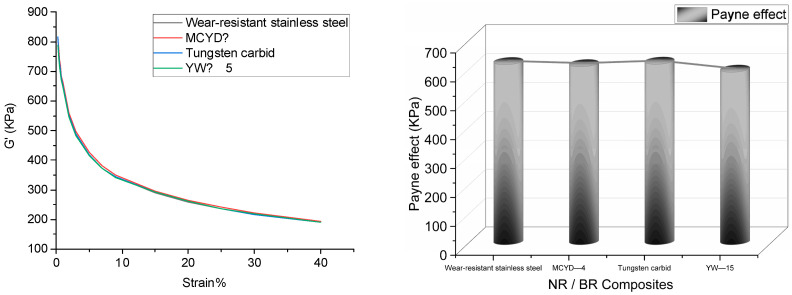
Payne effect of NR/BR composites after friction with different metals.

Figure 4 shows the stress–strain curve of NR/BR composites after friction with different metals, and the Payne effect of NR/BR composites is shown in Table 5.

The data was extracted from Figure 4 into Table 5. As shown in Table 5, the Payne effect of NR/BR composites was the highest after friction with tungsten carbide alloy, and the Payne effect of NR/BR composites was the lowest after a clash with YW 15 alloy. The Payne effect of the same NR/BR composites was different after friction with other metals, which mainly depended on the wear resistance of the metal. When NR/BR composites were rubbed with metal, the small metal particles on the metal surface were worn off due to abrasive wear caused by rubber. The filler dispersion was affected by incorporating small metal particles into the compound. Due to the different wear resistance of other metals, the number of small metal particles lost after friction with NR/BR composites differed. Generally speaking, the more the number of small metal particles, the more serious the obstruction of SiO_2_ dispersion in NR/BR composites. Therefore, it could be seen from the Payne effect that the NR/BR composites had the smallest metal particles after friction with tungsten carbide alloy, and the NR/BR composites had the tiniest small metal particles after a clash with YW-15 alloy. At the same time, the Payne effect of NR/BR composites had a slight difference after friction with four metals, which was consistent with the actual production situation.

#### 4.1.2. Tensile Tearing Performance of NR/BR Composites Rubbed with Different Metals

**Figure 5:**
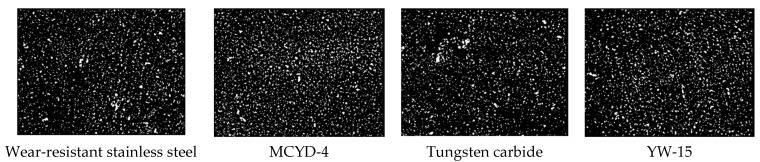
Image of dispersion of NR/BR composites after friction with different metals.

As can be seen from Figure 5 and Table 6, the dispersion of NR/BR composites was the highest after rubbing with YW-15 alloy, and the distribution of NR/BR composites was the lowest after rubbing with tungsten carbide alloy, which corresponds to the Payne effect of NR/BR composites.

### 4.2. Tensile Tearing Performance of NR/BR Composites Rubbed with Different Metals

**Figure 6:**
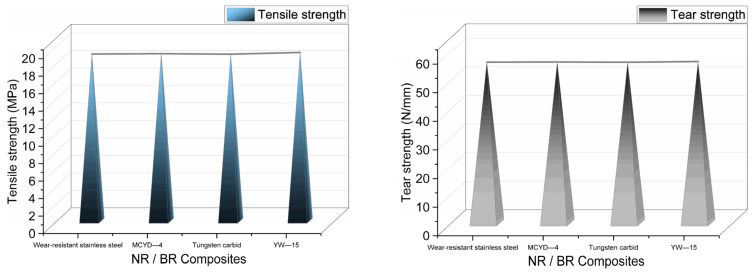
Tensile strength and tear strength of NR/BR composites after friction with different metals.

It can be seen from Figure 6 that the tensile strength and tear strength of NR/BR composites showed the same trend after friction with different metals. The tensile and tearing strength of the same NR/BR composites was additional after friction with other metals, related to the small metal particles in the friction process. The better the wear resistance of the metal was, the more minor the metal wear during the friction process, the less the tiny metal particles in NR/BR composites, and the smaller the change of tensile strength and tear strength of NR/BR composites. The NR/BR composites had the highest tensile strength and tear strength after friction with YW-15 alloy, which indicated that the metal wear amount of YW-15 alloy after friction was small, the number of small metal particles in the NR/BR composites were small, and the use of YW-15 alloy had a minor effect on the properties of NR/BR composites.

### 4.3. Dynamic Mechanical Properties of NR/BR Composites after Friction with Different Metals

**Figure 7:**
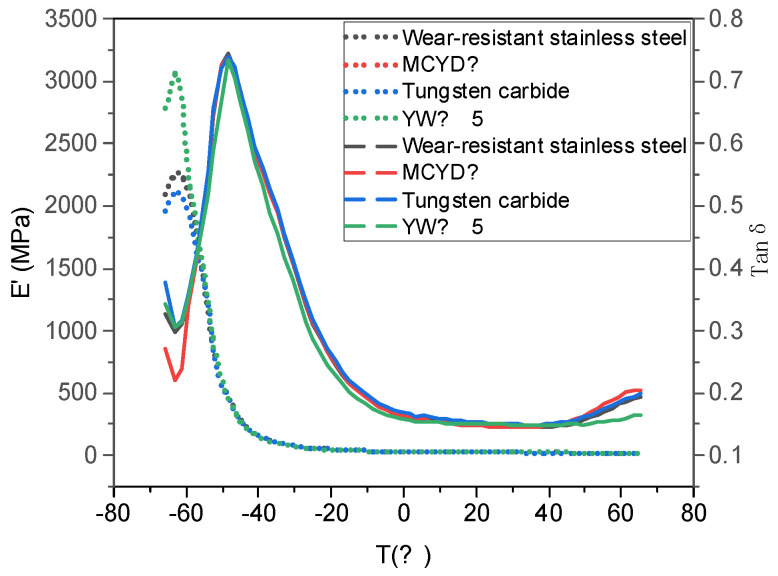
DMA test curve.

Figure 7 shows the curves of E’ and the loss factor (Tanδ) of NR/BR composites after friction with different metals as a function of temperature. The temperature corresponding to the maximum value of the Tanδ curve is usually used to characterize the rubber industry’s glass transition temperature (TG). TG can reflect the low-temperature resistance of NR/BR composites. Tanδ at different temperatures is commonly used to measure the dynamic mechanical properties of tires. Generally, Tanδ at 0 °C predicts the wet slip resistance of rubber materials, and at 40 °C and 60 °C represents the rolling resistance.

Figure 7 shows the effect of Tan δ and E’. The data in Figure 7 are extracted into Table 7. It can be seen from Table 7 that the glass transition temperature of the NR/BR composites after friction with YW-15 alloy was the lowest, and that of the NR/BR composites after friction with tungsten carbide alloy was the highest. Still, the temperature difference between the two was slight. This indicates that the NR/BR composites, after friction with YW-15 alloy, have the best low-temperature resistance.

In contrast, the NR/BR composites, after friction with other alloys, have a slightly worse low-temperature resistance. At the same time, according to Table 7, it can be seen from the data of 0 °C Tan δ that NR/BR after friction with YW-15 alloy has the best-wet slip resistance, and the wet slip resistance of NR/BR composites after friction with MCYD-4 alloy, wear-resistant stainless steel, and tungsten carbide alloy decreases gradually. From the value of tanδ at 40 °C and 60 °C, it can be known that the rolling resistance of NR/BR composites after friction with YW-15 alloy is the least, and that of NR/BR composites after friction with MCYD-4 alloy, wear-resistant stainless steel, and tungsten carbide alloy gradually increases.

This is related to the small metal particles in the compound which can increase the friction between the NR/BR composites and the ground and improve the rolling resistance of the NR/BR composites. At the same time, the small metal particles in the compound hinder the dispersion of SiO_2_ particles, so the more the tiny the metal particles are, the more SiO_2_ particle aggregates in the mix there are, which reduces the wet slip resistance of the NR/BR composites.

### 4.4. Silane Reaction Index

**Figure 8:**
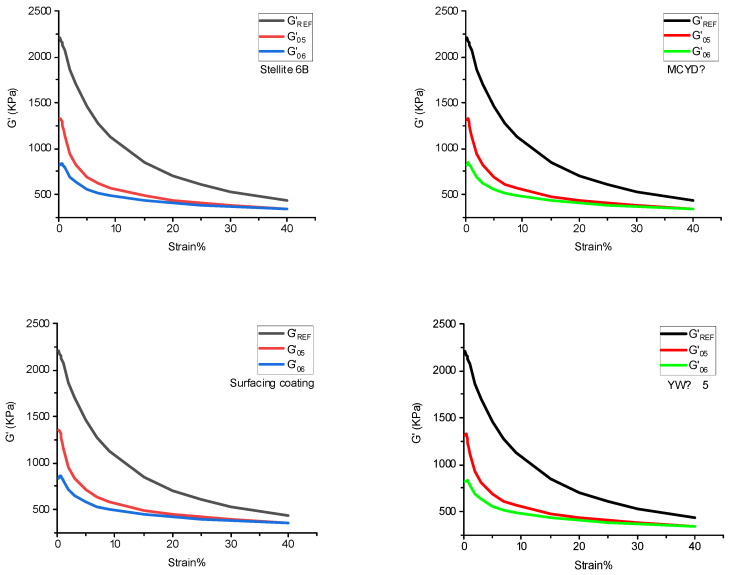
Degree of silanization reaction.

The degree of silanization reaction of NR/BR composites after friction with different metals is shown in Figure 8. The silanization reaction index is calculated according to the formula in 2.4.1, as shown in Table 8. According to the data in Table 8, there is a slight difference in the silanization reaction index of NR/BR composites after friction with four metals, which indicates that the degree of silanization reaction of NR/BR composites is related to metal. Still, metal has little influence on the degree of silanization reaction of NR/BR composites.

The internal mixer is sizeable industrial equipment, which is extremely difficult to disassemble, so the ethanol–water mixed vapor generated during the mixing process cannot be measured. In the field of rubber, the silanization reaction index is commonly used to characterize the degree of silanization reaction, and the proportion of silanization reaction index can qualitatively describe the balance of silanization reaction products. To accurately simulate the mixing process of the inner mixer, the high-temperature ethanol–water mixed vapor was sprayed onto the surface of the rubber compound and metal in proportion to the degree of silanization reaction (1:1.001:0.99:1.006) during the friction test on the CSM friction and wear testing machine.

### 4.5. CSM Friction and Wear Experiment

#### 4.5.1. The Average Friction Coefficient of NR/BR Composites after Friction with Different Metals

**Figure 9:**
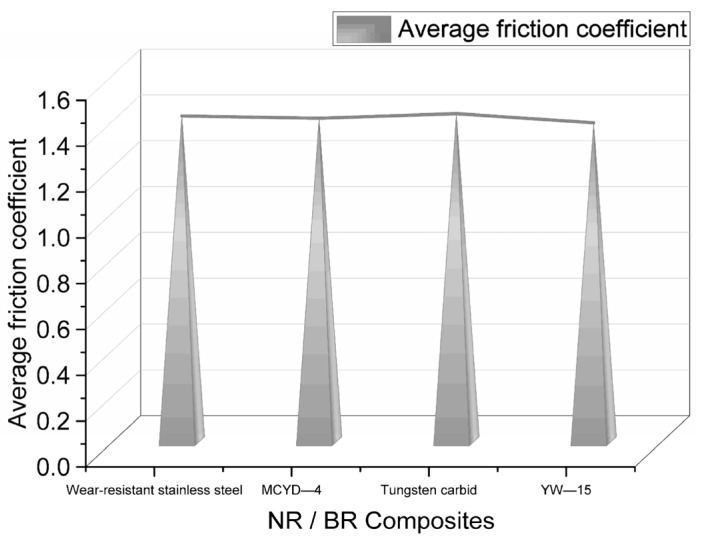
The average friction coefficient of NR/BR composites after friction with different metals.

Figure 9 shows the average friction coefficient measured by the CSM experiment.

The friction coefficient is mainly related to the dispersion of SiO_2_ particles in NR/BR composites and small metal particles. Generally speaking, the better the distribution of SiO_2_ particles, the smaller the number of small metal particles, the smaller the friction of NR/BR composites on metal, and the smaller the friction coefficient. The average friction coefficient of the same NR/BR composite material with different metals is different, which mainly depends on the wear resistance of the metal. After the friction of NR/BR composites with metal, the small metal particles on the metal surface are worn off due to abrasive wear caused by rubber. The filler dispersion is affected by incorporating small metal particles into the compound. Therefore, due to the different wear resistance of other metals, the number of small metal particles lost after friction with NR/BR composites is different. Generally speaking, the higher the number of small metal particles, the more serious the obstruction of SiO_2_ dispersion in NR/BR composites. Therefore, it can be seen from Figure 9 that the NR/BR composites have the most metal particles after friction with the tungsten carbide alloy, and the NR/BR composites have the smallest metal particles after friction with the YW-15 alloy.

#### 4.5.2. 3D Morphology of the Metal Grinding Head after Friction between NR/BR Composites and Different Metals

**Figure 10:**
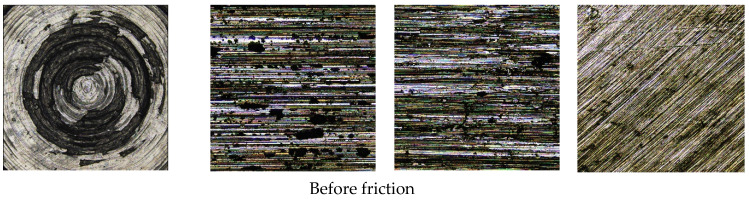

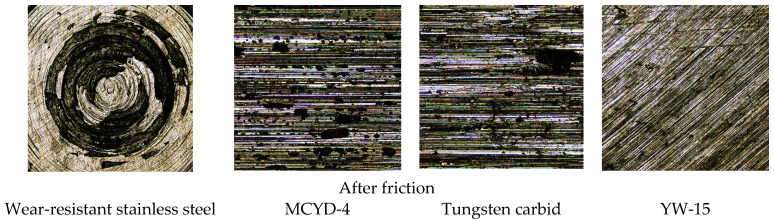
Surface morphology of different metal grinding heads before and after the friction with NR/BR composites (the width of one image is 1272 um).

**Figure 11:**
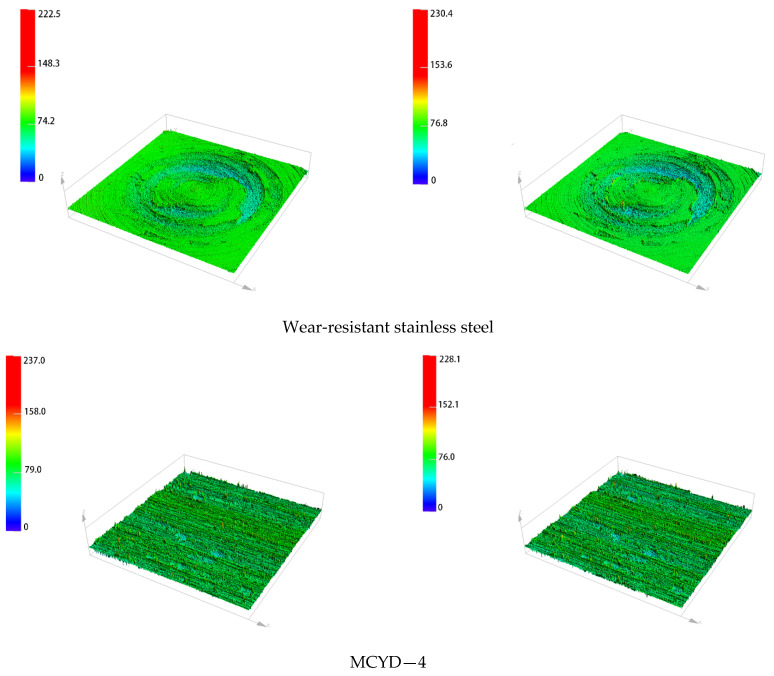

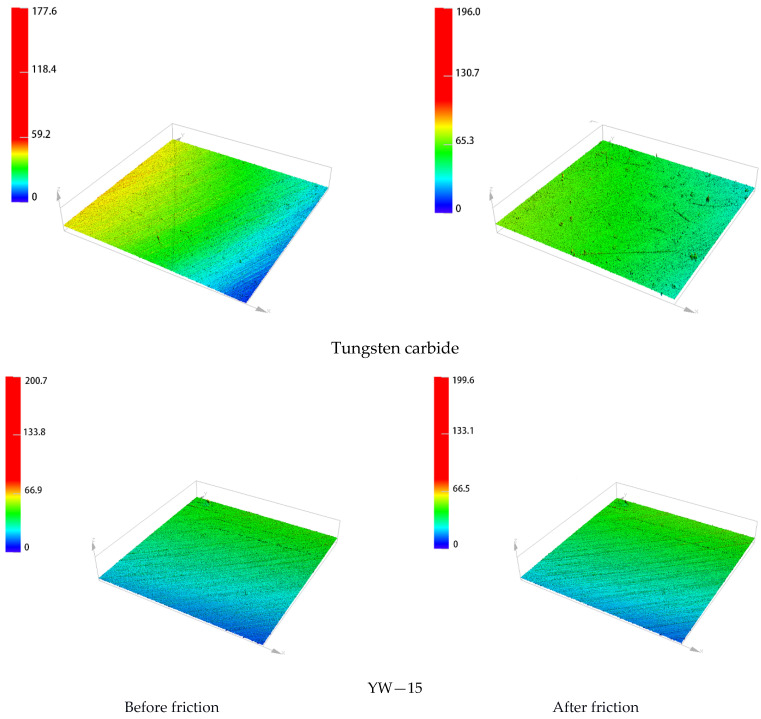
Three-dimensional morphology of metal surface before and after friction (using a tenfold magnifying glass).

Figure 10 shows the surface morphology of the metal grinding head before and after friction. Figure 11 shows the three-dimensional surface topography of the metal grinding head. As can be seen from Figure 10 and Figure 11**,** there were many scratches on the surfaces of the four metals after friction, and the pits on the surface of the tungsten carbide alloy were significantly expanded after friction.

#### 4.5.3. Height Profiles before and after the Friction between Different Metals with NR/BR Composites

**Figure 12:**
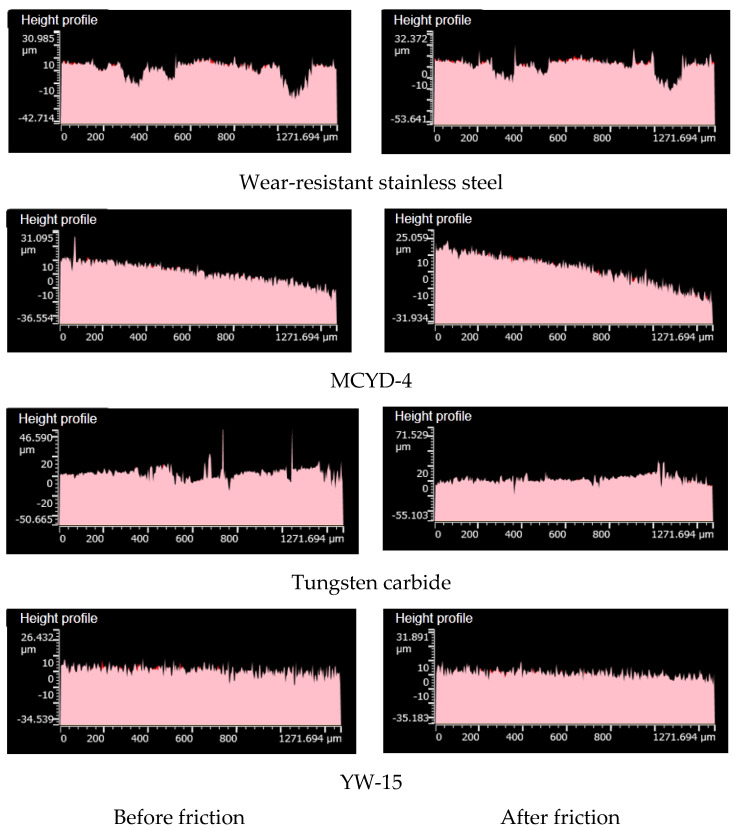
Height profiles before and after the friction between different metals with NR/BR composites.

Figure 12 shows the height profile before and after the friction between different metals and NR/BR composites. As demonstrated in Figure 12, after friction between YW-15 alloy and NR/BR composites, the height profile of the metal surface changes the least, indicating that YW-15 alloy has the best wear resistance. After the friction between MCYD-4 alloy or wear-resistant stainless steel and NR/BR composites, the height profile of the metal changes little, and only a few contour peaks are smoothed, which indicates that the wear-resistant of MCYD-4 alloy and wear-resistant stainless steel is relatively good. After the friction between tungsten carbide alloy and NR/BR composites, the height profile of the metal changes considerably; many contour peaks are ground flat, and the height profile changes before and after friction, which shows that the wear resistance of tungsten carbide alloy is worse than the other four alloys.

#### 4.5.4. Change of Metal Volume before and after Friction between Different Metals and NR/BR Composites

**Figure 13:**
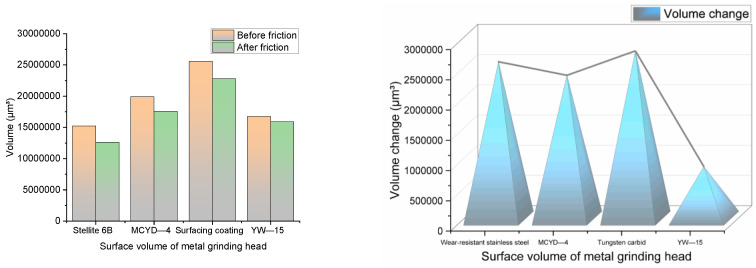
The change of metal volume before and after friction between different metals and NR/BR composites.

The metal volume changes before and after the friction between different metals and NR/BR composites are shown in Figure 13.

It can be seen from Figure 13 that the metal wear amount of YW-15 alloy after friction with NR/BR composites is much lower than that of the other three metals. The difference in metal wear is minor after the friction between MCYD-4 alloy or wear-resistant stainless steel and NR/BR composites. The metal wear is the highest after the friction between tungsten carbide alloy and NR/BR composites. From the point of wear quantity, the wear resistance of YW-15 alloy is far better than the other three metals.

#### 4.5.5. Corrosion Resistance Analysis

This experiment required the preparation of several groups of rubber for the investigation, four of which were sprayed with high-temperature steam during the friction process with the metal. The amount of metal wear measured at this time is the amount of corrosion and abrasive wear. The amount of metal wear measured at this point is called V1. Another group of rubber specimens was taken for CSM friction wear experiments, and this group of experiments did not spray a high-temperature ethanol-water steam mixture, so the measured wear amount was the abrasive wear amount of the metal. The abrasive wear measured in this group of experiments was recorded as V2.

Since the measurement of the experiments has errors, six repetitions were conducted to ensure the accuracy of the experiments, and the average value was taken for the calculation. After several repetitions of the investigation, the difference between V1 and V2 is the amount of corrosive wear. The amount of corrosion wear was recorded as V3.

Based on the above experimental data, the ratio occupied by the two wear methods can be calculated. Abrasive wear ratio = V2/V1.Corrosion wear ratio = V3/V1.

**Figure 14:**
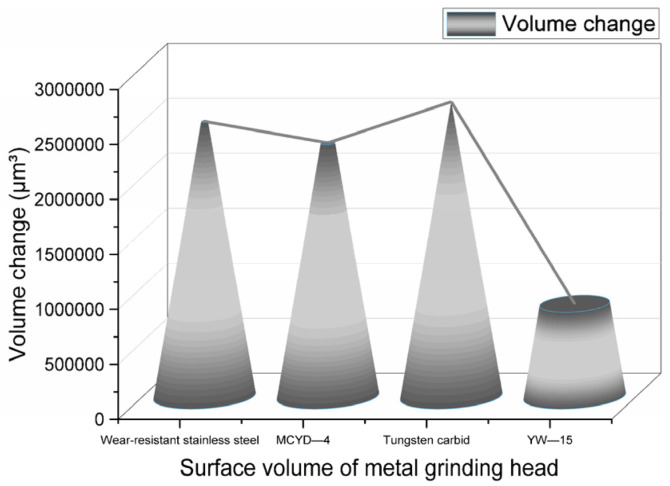
Metal wear volume without spraying high temperature ethanol–water vapor mixture.

Another rubber sample group was taken for the CSM friction and wear experiment. High-temperature ethanol and water mixed vapor was not sprayed in this experiment, so the wear quantity measured in this experiment was the abrasive wear quantity of metal. The metal volume changes before and after friction in this group of experiments are shown in Figure 14.

**Figure 15:**
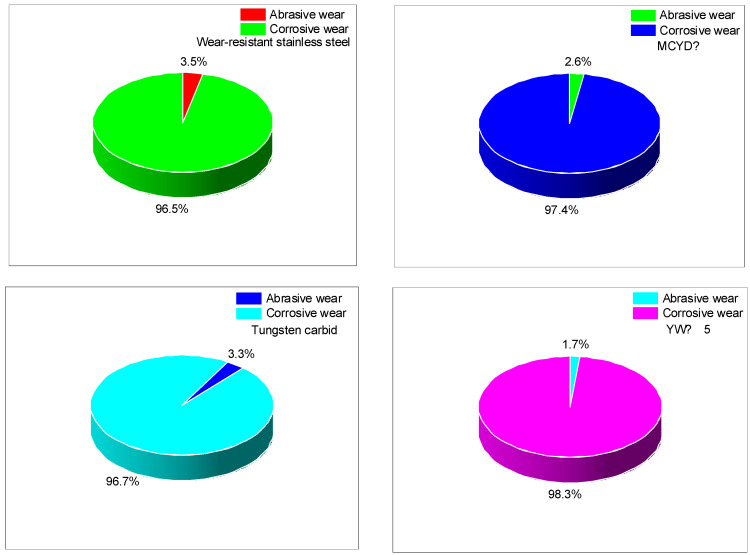
Proportion of abrasive wear and corrosion wear.

The abrasive wear and corrosion wear of metal can be calculated by comparing the wear measured in the experiment with that measured by spraying a high-temperature ethanol–water vapor mixture. To ensure the accuracy of the experiments, six repeated experiments were carried out, and the average value was calculated. The proportion of the two wear forms is shown in Figure 15.

Figure 15 can reflect the corrosion resistance of the four alloys to a certain extent. Taking the metal of the end face as the only variable, there are different amounts of small metal particles in NR/BR composites due to the metals’ differences in wear resistance. These small metal particles hinder the dispersion of SiO_2_ particles, thus affecting the silanization reaction. However, because the metal of the end face is the only variable and the experiment time is short, the product quality of the silanization reaction in the mixing process has little difference. Therefore, the corrosion resistance of the four alloys can be analyzed by comparing the proportion of corrosion and wear in Figure 15. The corrosion resistance of wear-resistant stainless steel and tungsten carbide alloy is the worst, and the ratio of corrosion wear is higher in the wear process. The corrosion resistance of YW-15 alloy and MCYD-4 alloy is better, and the proportion of corrosion wear of YW-15 alloy is only 1.7%.

#### 4.5.6. Changes of Metal Surface Roughness before and after Friction between Different Metals and NR/BR Composites

**Figure 16:**
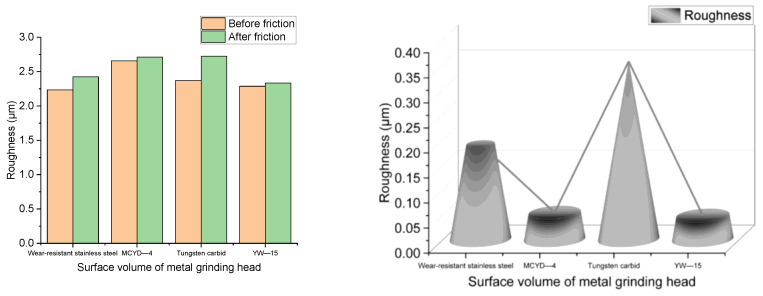
Roughness of metal surface before and after friction between different metals and NR/BR composites.

Figure 16 shows the changes in metal surface roughness before and after the friction between different metals and NR/BR composites.

It can be seen from Figure 16 that the roughness of the metal surface of YW-15 alloy and MCYD-4 alloy has little change after friction with NR/BR composites, and the roughness of the metal surface of wear-resistant stainless steel and tungsten carbide alloy changes significantly after friction with NR/BR composites. The surface roughness of the tungsten carbide alloy changes the most after friction with NR/BR composites. The change of metal surface roughness is mainly related to SiO_2_ particle aggregates and small metal particles. The better the wear resistance of the end face metal, the less the tiny metal particles doped in NR/BR composites, the less the resistance of the small metal particles to the dispersion of SiO_2_ particles, the less the number of SiO_2_ particle aggregates in NR/BR composites, the smaller the surface roughness change after the friction between metals and NR/BR composites. This means that the shift of metal surface roughness is directly proportional to the wear resistance of the metal. Figure 16 also intuitively reflects the wear resistance of the four alloys.

## 5. Conclusions

The wear and corrosion resistance of the four metals in the mixing process was studied in depth through the design of comparative experiments, which provided data support for selecting the end face metal of the inner mixer. 

(1) It was found that the tensile tear performance, wet slip resistance, and rolling resistance of NR/BR composites were different when different metals were chosen for the end face, but the difference was slight. When the end face metal was YW-15 alloy, NR/BR composites had the best dispersibility, the most robust tensile tear performance, the best-wet slip resistance, and the least rolling resistance. The physical and mechanical properties of NR/BR composites were reduced in different amplitudes when the end face metal was any of the other three kinds of alloys. This was related to the wear resistance of the metals. The higher the wear resistance of the metal, the fewer the tiny metal particles in the NR/BR composites. The small metal particles hindered the dispersion of SiO2 particles. The fewer the tiny metal particles, the better the distribution of SiO2 particles was, and the better the performance of NR/BR composites was. 

(2) It was also found that YW-15 alloy had the best wear resistance and the most robust corrosion resistance. The wear resistance and corrosion resistance of wear-resistant stainless steel and tungsten carbide alloy were relatively poor. Corrosion wear was still an essential factor that could not be ignored in the mixing process.

## Figures and Tables

**Table 1:** Experimental instruments and equipment.

Instruments and Equipment	Manufacturer
Hake mixer	Qingdao University of Science and Technology, Qingdao, China
BL-6157 roller crusher	Baolun Precision Testing Instrument Co., Dongguan, China
3D laser measuring microscope (LEXT OLS5000)	Olympus Corporation, Tokyo, Japan
carbon black dispersing meter (Disper GRADER)	Alpha Company, Wilmington, DE, USA
steam generator (ZT-2588S)	Zhiteng Company, Taiwan
rubber processing performance analyzer (RPA2000)	American Alpha Company, South Brunswick, NJ, USA
CSM-Friction and wear testing machine	Tribometer company, Switzerland

**Table 2:** Materials.

Raw Material	(phr)
BR9000	25.5
NR	15
N234	25
Silica115MPa	30
C5	4
4020	5
H7075	2
ZnO	3
SAD	2
TESPT	6
DPG	0.18
S	2.8
CZ	1.8

**Table 3:** Mixing process of mixed rubber.

Hake Mixer		
Time	T (°C)	Ingredients
BR9000 + NR		
0:00	40	Polymers
0:35		Chemical, 1/2 Silica115MP
1:35		1/2 Silica115MP
2:25	120	Sweep
3:55	135	Sweep, Sampleing
5:00	145	Discharge

**Table 4:** RPA method is used to measure the degree of a silanization reaction.

Stage	Frequency/Hz	T/°C	Time/min	Strain	Test Items
1	0.1	60	5	0.28%	-
2	1	60	-	0.28–40%	*G*′(02)
3	1	60	-	0.28–40%	*G*′(03)
4	0.1	60/160/160	0/2.5/5	0.28%	-
5	1	60	-	0.28–40%	*G*′(05)
6	1	60	-	0.28–40%	*G*′(06)

**Table 5:** Payne effect of NR/BR composites after friction with different metals.

Metals	Wear-Resistant Stainless Steel	MCYD-4	Tungsten Carbide	YW-15
Payne effect	623.33	616.07	624.65	597.14

**Table 6:** Dispersion value of NR/BR composites after friction with different metals.

NR/BR Composite	Wear-Resistant Stainless Steel	MCYD-4	Tungsten Carbide	YW-15
Dispersion	6.17	6.45	6.15	6.75

**Table 7:** TG and Tanδ of NR/BR composites after friction with different metals.

DMA	Wear-Resistant Stainless Steel	MCYD-4	Tungsten Carbide	YW-15
TG(°C)	−47.029	−47.0889	−46.5976	−47.1629
0 °Ctanδ	0.163911	0.165656	0.15913	0.170153
40 °Ctanδ	0.149516	0.147592	0.150543	0.144879
60 °Ctanδ	0.186308	0.183055	0.194639	0.157781

**Table 8:** Silanization reaction index of NR/BR composites after friction with different metals.

NR/BR Composites	Wear-Resistant Stainless Steel	MCYD-4	Tungsten Carbide	YW-15
Silanization reaction index	0.86	0.861	0.856	0.865

## Data Availability

My data is accurate and reliable, and the experiment can be repeated. I promise that my information is fully available.
